# Associations between cognitive and clinical functioning and AD blood based biomarkers among Hispanic and WNH older adults

**DOI:** 10.1002/alz70856_101390

**Published:** 2025-12-25

**Authors:** Miriam J. Rodriguez, Batul Yawer, Patricia Garcia, Michael Marsiske, Ranjan Duara

**Affiliations:** ^1^ Indiana University School of Public Health, Bloomington, IN, USA; ^2^ University of Florida, Gainesville, FL, USA; ^3^ Indiana University School of Medicine, Indianapolis, IN, USA; ^4^ Florida Alzheimer's Disease Research Center, Gainesville, FL, USA; ^5^ Wien Center for Alzheimer's Disease and Memory Disorders, Miami Beach, FL, USA; ^6^ Mount Sinai Medical Center, Miami Beach, FL, USA; ^7^ 1Florida Alzheimer's Disease Research Center, Miami, FL, USA

## Abstract

**Background:**

We examined and compared the associations of Alzheimer's disease (AD) blood‐based biomarkers to clinical and cognitive functioning in Hispanic (*n* = 214) (an understudied group) and White‐non‐Hispanic (WNH) (*n* = 169) older adults.

**Methods:**

Neuropsychological and Functional assessments were completed and AD blood biomarkers were obtained in cognitively normal (*n* = 62), PreMCI (*n* = 36), MCI (*n* = 217), or dementia (*n* = 68) subjects. Functional limitations were measured by the modified Clinical Dementia Rating (mCDR). Ordinary least squares models were conducted using unit‐weighted composites for the dependent blood biomarker variables ptau217, abeta40, abeta42, GFAP, and NFL concentrations, controlling for age, sex, education, ethnicity, and cognitive diagnosis.

**Results:**

After inclusion of covariates, we examined unstandardized (b) and standardized regression weights (stb) for all three biomarkers among both ethnic groups (Hispanic and WNH). In the Hispanic group, higher levels of ptau217 concentration predicted lower memory scores (b=‐4.24, stb=‐0.26, *p* = 0.002), and lower word fluency scores (b=‐3.48, stb=‐0.22, *p* = 0.016). Higher NFL concentration significantly predicted lower word fluency scores (b=‐0.15, stb=‐0.2, *p* = 0.047) and higher functional limitation scores (b= 0.27, stb = 0.36, *p* =  0.00). Education predicted Trails, letter fluency, and word fluency. In the WNH group higher Abeta40 (b=‐0.05, stb= ‐0.36, *p* = 0.048) and GFAP (b =‐0.03, stb=‐0.35, *p* = 0.024) concentrations predicted lower letter fluency scores, and higher ptau217 (b=5.79, stb=0.30, *p* = 0.029) and lower abeta42 concentrations (b=1.05, stb=0.35, *p* = 0.043) were predictors of higher letter fluency scores. Significant standardized regression weights (‐0.36 to 0.35) of biomarkers had small to moderate unique effect sizes.

**Conclusion:**

Blood‐based biomarker levels were found to be associated with cognitive functioning among Hispanic and WNH older adults and associations to functional limitations were present only in the Hispanic group. Associations of Ptau217 with memory, word fluency, and associations of NFL with functional limitations appeared to have a larger effect among Hispanics. While Abeta 40, abeta42, GFAP, and NFL were associated with letter fluency only among WNH. Our results indicate that the use of blood‐based biomarkers may be predictors of cognitive status and functional impairment among Hispanic ADRD populations. This may enhance diagnostic accuracy and address health disparities among this population.

